# Preparation, Characterization and Adsorption Performance of a Novel Anionic Starch Microsphere

**DOI:** 10.3390/molecules15042872

**Published:** 2010-04-21

**Authors:** Yati Yang, Xiuzhi Wei, Peng Sun, Juanmin Wan

**Affiliations:** 1 College of Science, Northwest Agriculture & Forest University, Yangling, 712100, Shanxi, China; E-Mail: weixiuzhi@nwsuaf.edu.cn (X.W.); 2 College of Resources and Environment, Northwest Agriculture & Forest University, Yangling, 712100, Shanxi, China; E-Mail: juanminwuan@163.com (J.W.)

**Keywords:** inverse emulsion, anionic starch microspheres, adsorption, methylene blue

## Abstract

Neutral starch microspheres (NSMs) were synthesized by an inverse microemulsion technology with epichlorohydrin as a crosslinker and soluble starch as starting material. Anionic starch microspheres (ASMs) were prepared from NSMs by the secondary polymerization with chloroacetic acid as the anionic etherifying agent. Fourier transform infrared spectroscopy (FT-IR), scanning electron microscopy (SEM) and laser diffraction particle size analyzer were used to characterize the anionic starch microspheres. The results showed that structure of the microspheres was compact and the hardness of microspheres was great, and the average diameter of the product was about 75 µm. The anionic starch microspheres (ASMs) were used to adsorb methylene blue (MB) from aqueous solution. Effects of adsorption time, initial concentration of MB, and temperature on the adsorption of MB onto ASMs were studied, and the equilibrium and kinetics of the adsorption process were further investigated. It shows that ASMs can effectively remove MB from the solution. The adsorption equilibrium data correlates well with the Langmuir isotherm model compared with Frendlich isotherem model. The pseudo-first-order and pseudo-second-order kinetic models were applied to test the experimental data. The pseudo-second-order kinetic model provided a better correlation of the experimental data in comparison with the pseudo-first-order model. Temperature variations did not significantly affect the adsorption of MB onto ASMs.

## 1. Introduction 

In our modern industrial society, dyes are widely used to color products for textiles, printing, dyeing, and food. Synthetic dyes in an effluent, even in a small amounts, are highly visible and can have undesired effects, not only on the environment, but also on living creatures. In addition, some dyes and their degradation products may be carcinogenic and toxic, consequently, they are important sources of water pollution and their treatment becomes a major problem for environmental managers. Adsorption is one of the most efficient methods to remove pollutants from wastewater. Many studies have been made on the possibility of using adsorbents based on activated carbon [1], clay minerals [2,3,4], crosslinked amphoteric starch [5], weeds [6], fly ash [7,8], Indian rosewood sawdust [9], and cross-linked chitosan beads [10]. However, the adsorption capacity of these adsorbents is not very large, so new absorbents are still under development to improve adsorption performance.

Starch and its derivatives represent a cheap and environmentally safe source of raw material for the preparation of low-cost adsorbents that may be useful for the removal of pollutants from wastewater. This biopolymer represents an interesting alternative as an adsorbent because of its particular characteristics (abundant, renewable and biodegradable raw resource) and properties such as its chemical stability and high reactivity, resulting from the presence of chemically reactive hydroxyl groups in its polymer chains [11], which make it possible to chemical modify starch according to different requirements. Among various modifications, crosslinked starch microspheres show high stability towards swelling, high temperature, high shear and acidic conditions [12], and have been the most investigated drug carriers and adsorbents due to their total biodegradability, biocompatibility, nontoxicity, stability on storage, cost effectiveness as well as simple fabrication method [13]. The majority of studies have focused on starch microspheres, cellulose-based matrix microspheres, sodium carboxymethylcellulose polymers and chitosan-based microparticles as drug delivery systems [14,15,16,17] and there are only a few reports about the use of starch microspheres as adsorbent. After modification, starch microspheres have suitable expansion, huge pore volume and high specific area, which can enhance its adsorption ability. It means that modified starch microspheres are more suitable as adsorbent for dyes and heavy metals or as catalyst carrier materials. Therefore the preparation of nontoxic, renewable, low cost ionized starch microspheres is not only of importance in the field of modern pharmacy but also has great prospects in the water treatment field.

In recent years, many research works have covered the preparation of neutral starch microspheres and their physicochemical properties [18]. Common neutral starch microspheres mainly adsorb physically, so its adsorption and selectively adsorption ability are weak. Ionization of starch microspheres can enhance the active groups and thus improve its adsorption ability. Anionic starch microspheres have high affinity to positively charged drugs, dyes, metals ion, thus enhancing the selective adsorption performance. However, until now a few works have been done on anionic starch microspheres [19], and no work has covered the adsorption of anionic starch microspheres containing carboxyl groups. It has been reported [20] that introduction of reactive functional groups in the backbone of highly crosslinked starches gave products that were capable of removing heavy metal ions from industrial wastewater. 

In this study, crosslinked starch microspheres were synthesized by an inverse emulsion technology with epichlorohydrin as a crosslinker and soluble starch as starting material. Then chloroacetic acid was used as the anionic etherifying agent to prepare anionic crosslinked starch microspheres. The morphology of the starch microspheres was examined through Scanning Electron Microscopy (SEM) and Fourier Transform InfraRed spectroscopy (FTIR) was used to study the structural organization of anionic starch microspheres. Methylene blue (MB) was employed as a model basic dye. A thermodynamic investigation and kinetic study were employed to research the mechanism of adsorption of MB in solution by anionic starch microspheres.

## 2. Results and Discussion

### 2.1. Morphology of ASMs

**Figure 1:**
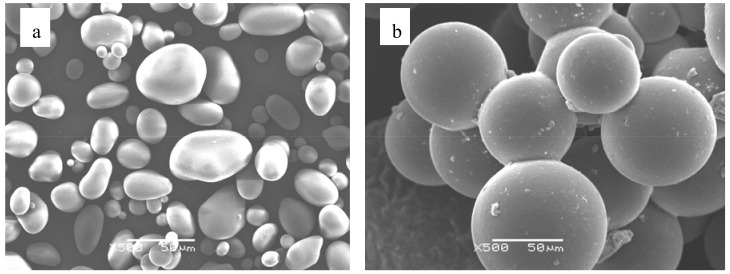
Scanning electron micrograph (SEM) of (a) soluble starch; (b) ASMs.

Figure 1 shows the morphology of soluble starch and ASMs. The soluble starch particles are irregular and of different size. ASMs exhibited smooth surface and spherical shape, but they had a high size polydispersity, and were joined to other ASMs forming aggregates. It is estimated that the aggregation was mainly caused by the emulsification method involved. When emulsified by mechanical stirring, the size distribution of the droplets in the emulsion was wide, thus the coalescence and breakup of droplets occurred frequently during the emulsification and crosslinking process. Consequently, the adhesion between microparticles cannot be avoided [21].

### 2.2. Structural Organization by FTIR

**Figure 2:**
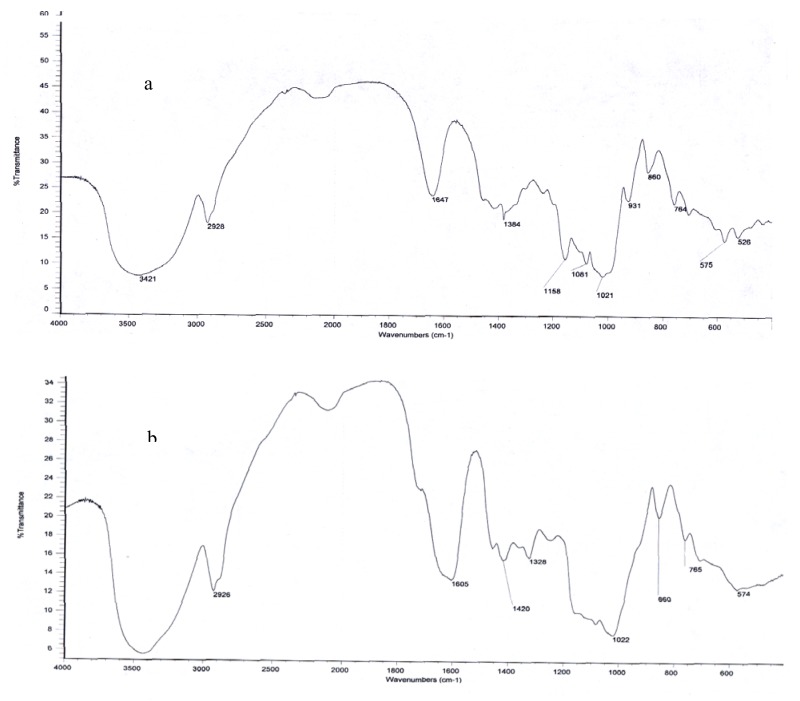
FTIR spectra of (a) soluble starch; (b) ASMs.

The structural organization of ASMs was further investigated by FTIR. The FTIR spectra of soluble starch and ASMs are shown in Figure 2. It is obvious that there are significant differences between the spectrum of soluble starch and that of the ASMs. In the region of 3,400 cm^−1^ and 2,920 cm^−1^, the spectrum of soluble starch and ASMs both showed broad and strong bands. It means that hydroxyl groups exist before and after crosslinking, but it is clear that ASMs’ band is less broad than that of soluble starch. Maybe it is because that crosslinking and carboxylation decrease the hydrogen bond connections. The spectrum of soluble starch showed six major discernible bands located at 1,647, 1,384, 1,158, 1,081, 1,021, and 931 cm^−1^. Among them, the band at 1,081 cm^−1^ could be observed as a sharp band, and the band at 1,021 cm^−1^ was weak. At the same region, the spectrum of ASMs showed significant changes in comparison with that of soluble starch. The bands at 1,158, 931 cm^−1^ became indiscernible and the band at 1,081 cm^−1^ became week, while the band at 1,022 cm^−1^ seemed to be better resolved and predominant. It is well known that the bands at 1,081 and 1,022 cm^−1^ are sensitive to changes in crystallinity. The band at 1,081 cm^−1^, which is associated with the amount of ordered or crystalline starch, increases with increasing crystallinity, and the band at 1,022 cm^−1^, which is characterisic of amorphous starch structures increase with decreasing crystallinity [22]. Because of crosslinking and anionic, the crystallinity of soluble starch decreased, which correlates well with the changes observed in the bands located at 1,081 and 1,022 cm^−1^. It is noteworthy that new bands appeared at 1,605 cm^−1^ and 1,420 cm^−1^ in the spectrum of ASMs which are the characteristic carboxylate absorbtion bande [23]. All these change in the spectrum indicates that the anionic starch microspheres were synthesized successfully.

### 2.3. Particle Size of ASMs

**Figure 3:**
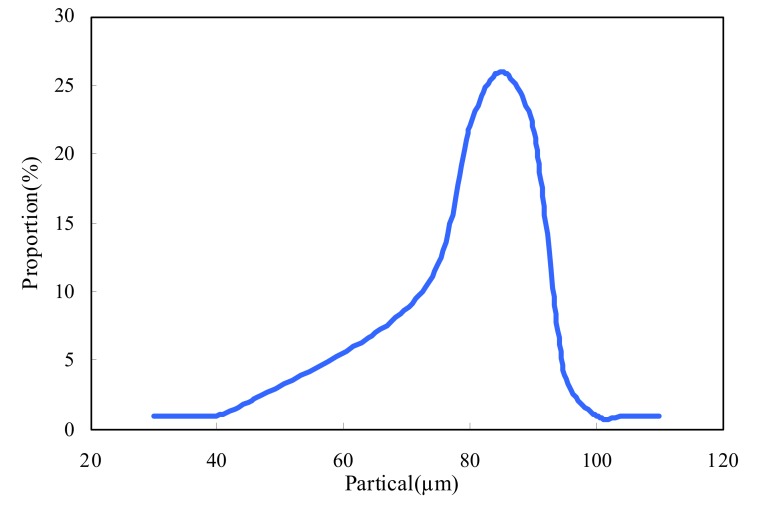
Particle size distribution of ASMs.

The size distribution of ASMs could be seen in Figure 3. Over ninety percent of the microspheres were smaller than ninety micrometers.

### 2.4. The Adsorption Isotherm and Kinetic Characteristics of MB Adsorption on Microspheres

#### 2.4.1. Effect of Initial MB Concentration

**Figure 4:**
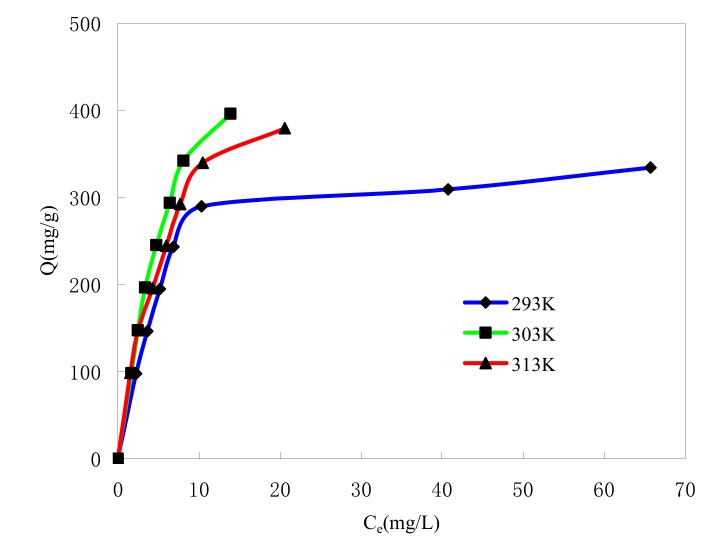
Effect of initial MB concentration on MB adsorption by ASMs. t = 2 h.

Figure 4 shows the effect of the initial MB concentration on the adsorption. The Langmuir and Freundlich isotherm models [24] were used to study the adsorption isotherm. The Langmuir adsorption isotherm can be expressed as a simple model (1) in which the attachment of adsorbate to the surface is represented by:

(1) $$\frac{C_{e}}{Q_{e}}=\frac{1}{Q_{m}b}+\frac{C_{e}}{Q_{m}}$$

where C_e_ is the equilibrium concentration of MB in the solution (mg/L), Q_e_ is the adsorption amount at the equilibrium (mg/g), Q_m_ is the maximum capacity (mg/g) and b is the Langmuir constant related to the affinity of binding sites (L/mg). The straight lines were given by plotting C_e_/Q_e_ *versus* C_e_, which give the values of b and Q_m_ according to the intercept and the slope of line. Table 1 lists the calculated results.

The Freundlich adsorption isotherm (2) is also used to fit the experimental data:

(2) $${\mathrm{lnQ}}_{e}=\ln K_{F}+\frac{1}{n}\ln C_{e}$$

where K_F_ is roughly an indicator of the adsorption capacity, 1/n is of the adsorption intensity. K_F_ and 1/n can be determined from the linear plot of lnQ_e_ *versus* lnC_e_ according to the intercept and the slope of line. Table 1 lists the calculated results. The magnitude of the exponent 1/n gives an indication of the favorability of adsorption. The value of n is more than 1, which represent favorable adsorption condition [25].

The free energy change (△G^Ө^) was calculated using the relationship below and is listed in Table 1:

$$\Delta G^{\theta }=-RT\mathrm{lnbM}$$

where R is the gas constant(J/mol·K), T is the temperature(K). M is the molecular weight of MB(mg/mol). And b is the Langmuir constant related to the affinity of binding sites (L/mg).

The correlation coefficients (R^2^ > 0.95) in Table 1 show that the adsorption isotherm can be explained using both the Langmuir and Freundlich models. The values of the Freundlich correlation coefficient (R^2^) are lower than the Langmuir values, which show that the Langmuir equation represents a better fit of experimental data than the Freundlich equation. The good applicability of the Langmuir isotherms to MB adsorption shows that both monolayer adsorption (chemical sorption) and homogeneous distribution of active groups on the surface of the adsorbent are possible [26]. Table 1 also indicates that the calculated maximum monolayer capacity Q_m_ of MB onto ASMs has a large value (Q_m_ > 350 mg/g), which is much higher than that of neutral microspheres used to adsorb MB [18]. Thermodynamic considerations tell us that at constant temperature and pressure, free enthalpy change (ΔG^Ө^) during the spontaneous adsorption process always has a negative sign. In Table 1, negative ΔG^Ө^ values showed the adsorption of MB onto ASMs is spontaneous.

The correlation coefficients (R^2^ > 0.95) in Table 1 show that the adsorption isotherm can be explained using both the Langmuir and Freundlich models. The values of the Freundlich correlation coefficient (R^2^) are lower than the Langmuir values, which show that the Langmuir equation represents a better fit of experimental data than the Freundlich equation. The good applicability of the Langmuir isotherms to MB adsorption shows that both monolayer adsorption (chemical sorption) and homogeneous distribution of active groups on the surface of the adsorbent are possible [26]. Table 1 also indicates that the calculated maximum monolayer capacity Q_m_ of MB onto ASMs has a large value (Q_m_ > 350 mg/g), which is much higher than that of neutral microspheres used to adsorb MB [18]. Thermodynamic considerations tell us that at constant temperature and pressure, free enthalpy change (ΔG^Ө^) during the spontaneous adsorption process always has a negative sign. In Table 1, negative ΔG^Ө^ values showed the adsorption of MB onto ASMs is spontaneous.

#### 2.4.2. Effect of Adsorption Time

To better understand the adsorption mechanism between ASMs and MB in aqueous solution, the adsorbed amounts of MB on ASMs were measured as a function of time.

**Figure 5:**
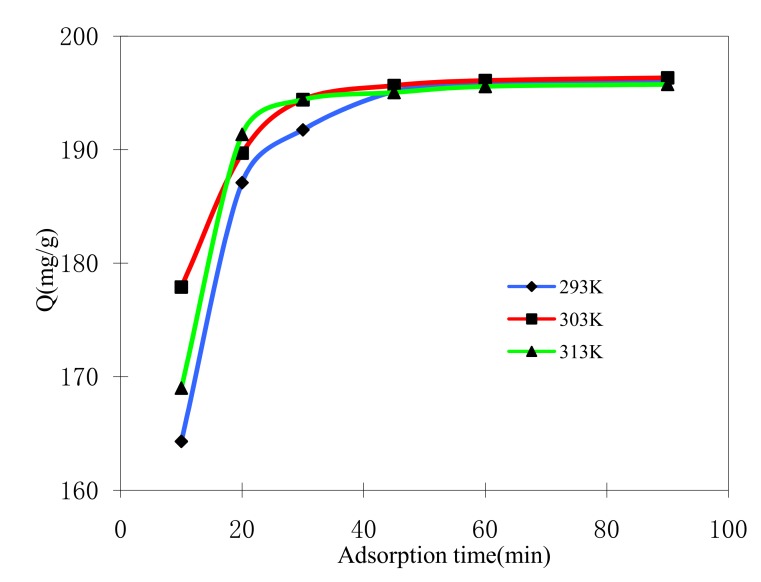
Effect of adsorption time on MB adsorption by ASMs.C_0_ = 200 mg/L.

As shown in Figure 5, MB is rapidly removed by ASMs and the adsorption processes reach equilibrium in about 30 min. Although the temperature of adsorption is different, the equilibrium time is almost the same. In order to investigate the mechanism of adsorption, two different models were used to investigate the experimental data of temperature for the adsorption process. The pseudo-first-order model (3) and pseudo-second-order model (4) are described as follows [7,8]:

(3) $$\ln\left(Q_{e}-Q_{t}\right)=\ln Q_{e}-K_{1}t$$

(4) $$\frac{t}{Qt}=\frac{1}{K_{2}Q_{e}^{2}}+\frac{1}{Q_{e}}t$$

where $Q_{e}$ and $Q_{t}$ (mg g^−1^) are the adsorbed amounts of MB on ASMs at equilibrium and at time t, respectively; K_1_ (min^−1^), K_2_ (g mg^−1^ min^−1^) are the rate constants for pseudo-first-order, pseudo-second-order. 

Adsorption kinetic parameters for three different temperatures are shown in Table 2. The values of correlation coefficient (R^2^) indicate a better fit of pseudo-second-order model with the experimental data compared with the pseudo-first-order model. Moreover, the equilibrium adsorption capacity calculated from the pseudo-second-order kinetic model fitting is nearer equilibrium adsorption amount (Qe-exp) from the experiment than the pseudo-first-order kinetic model fitting. It means that chemical adsorption is the determine step of the adsorption process rather than mass transfer in solution [27]. The similar phenomena are also observed in biosorption of dye RB2, RY2 and Remazol black B on biomass [10,28,29]. The ASMs in this study have relatively high equilibrium adsorption amount Q_e_, and the equilibrium time was very short. Such short equilibrium time coupled with high adsorption capacity indicate a high degree of affinity between the MB and the ASMs.

#### 2.4.3. Effect of Adsorption Temperature

**Figure 6:**
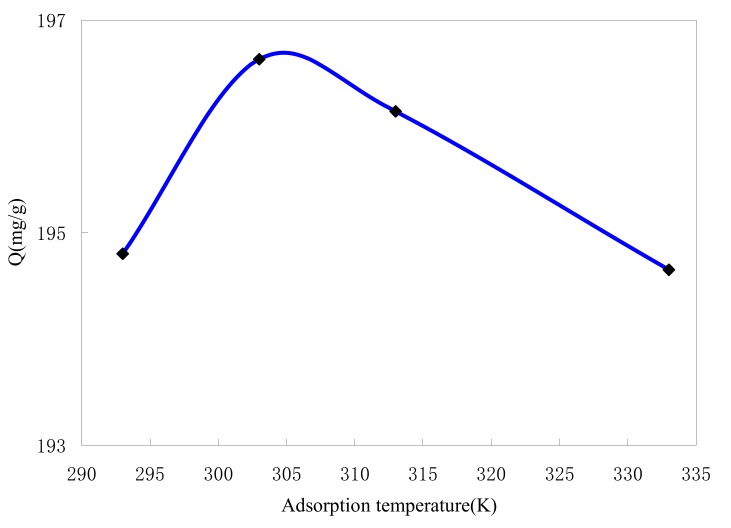
Effect of adsorption temperature on MB adsorption by ASMs.C_0_ = 200 mg/L, t = 2 h.

The effect of adsorption temperature on dye adsorption by ASMs was shown in Figure 6. The results show that there is no consistent trend found for the relationship between temperature and amount of adsorption.

According to Figure 6 an increase in the temperature leads to an increase in MB adsorption below 303 K, which indicates the adsorption is endothermic. Above 303 K, the decrease of adsorption capacity with increasing the temperature indicates that the adsorption of MB onto ASMs is controlled by an exothermic process. A similar temperature effect on the adsorption trend has also been shown in the cases of adsorption of methyl green onto crosslinked amphoteric starch [5], of RR 189 onto cross-linked chitosan beads [10], and of acid blue 25 onto peat [30].

A reasonable explanation for this would be that endothermic static interaction is dominant below 303 K, while the exothermic hydrophobic interactions become dominant above 303 K. So 303 K is the equilibrium point for these two interactions [31]. However, it can be seen from Figure 6 that these effects are insignificant. Normal wastewater temperature variations do not significantly affect the overall decolorization performance [32].

### 2.5. Comparison with other adsorbents

In order to justifying the validity of ASMs as an adsorbent of MB, its adsorption was compared with other various adsorbents reported in literature. Table 3 lists the comparison of maximum monolayer adsorption capacity of MB onto various adsorbents. It is clear that ASMs used in this work had a higher adsorption capacity of 666.67 mg/g compared to other adsorbents found in the literature.

## 3. Experimental 

### 3.1. Materials and Reagents 

Soluble starch (Tianjin Dibo Chemical Co.), sodium hydroxide (Xi’an Chemical Co.), epichloro-hydrin (Tianjin Chemical Co.), Span60 (GuoYao Chemical Co.), ethylacetate (Tianjin Dibo Chemical Co.), absolute alcohol (Xi’an Sanpu Chemical Co.), acetone (Tianjin Dibo Chemical Co.), chloroacetic acid (Chendu Lianhe Chemical Co.), hydrochloric acid (Chendu Kelong Chemical Co.), Silver Nitrate (Xi’an Chemical Co.). 

### 3.2. Synthesis of Anionic Starch Microspheres (ASMs)

#### 3.2.1. Preparation of Neutral Starch Microspheres (NSMs)

In order to preparing anionic starch microspheres (ASMs), neutral starch microspheres (NSMs) were synthesized first by an inverse emulsion. A solution of starch was prepared by dissolving the planned amount of soluble starch in a breaker which contained desired 2 mol/L sodium hydroxide solution, and then the starch solution was stirred for 30 min at 60 °C, then the starch solution was cooled to room temperature as the aqueous phase. Soybean Oil (70 mL) was mixed with the desired amount of Span60 under mechanical stirring at 60 °C until the Span60 was dissolved. After this the solution was cooled to 40 °C as the oil phase. Subsequently, the aqueous phase was added drop-wise to the oil phase under mechanical stirring to produce an inverse emulsion. After 30 min of emulsification, epichlorohydrin (4 mL) was added to the emulsion as cross-linking agent to develop microspheres. The solution was stirred for another 6 h at 40 °C with a constant stirring speed of 200 rpm. The microspheres were eventually centrifuged to remove oil and then washed in turn with ethyl acetate, absolute alcohol, and acetone. Finally, the microspheres were dried at 40 °C for 6 h [41].

#### 3.2.2. Preparation of Anionic Starch Microspheres (ASMs)

Neutral starch microspheres (1 g) were weighed in a flask, then 80% ethanol (4 mL), 20% sodium hydroxide solution (1.5 g) and chloroacetic acid (0.3 g) were added in turn. The mixture was reacted at 50 °C by shaking for 5 h. The reactor was eventually neutralized with 20% hydrochloric acid, filtered and washed with 80% ethanol until it gave a negative chlorine test with silver nitrate. The anionic microspheres were dried at 40 °C for 6h.

### 3.3. Measurements 

#### 3.3.1. Scanning Electron Microscopy (SEM)

The morphology of ASMs were studied in a JSM-6360LV SEM (Japan) operating at 25 kV. Microparticles for SEM studies were mounted on metal stubs with double-side adhesive, and coated with gold in vacuum using an IB-3ion coater (Eiko, Japan)

#### 3.3.2. Fourier Transform InfraRed (FTIR) spectroscopy

The FTIR measurements were performed in the solid state with a Nexus 470 FTIR machine (Nicolet, USA). The dry samples were crushed with KBr and pressed into pellets. Spectra were scanned in the range between 4,000 and 400 cm^−1^. Prior to recording, the spectra were recorded against a KBr background.

#### 3.3.3. Particle size analysis

The dry microspheres were dispersed in absolute alcohol and their particle size distribution was determined by a Mastersizer 2000 laser paricle analyzer (Malvern, UK). Volume distribution of ASMs samples was plotted using a computer program supplied by the manufacturer. The results were expressed in micrometers.

#### 3.3.4. Batch equilibrium studies

The adsorption experiments were carried out in a series of conical flasks containing 0.05 g of ASMs and 50 mL of methylene blue solution at the desired concentration in a water bath to control the temperature. After two hours shaking, the flasks were removed and the concentration of MB after the adsorption was analyzed at wavelength 665 nm by an UV-VIS spectrometer (Model 754, Shang Hai). The adsorbed amount of MB on microspheres was computed as follows:

(5) $$Q=\frac{\left(C0-C1\right)\times V}{m}$$

where Q is the amount of adsorbed MB (mg/g); C_0_ and C_1_ are the initial and equilibrium concentration (mg/L), respectively; V is the volume of MB aqueous solution (L); m is the dose of ASMs (g). All the experiments were repeated three times and the average values are presented in this communication.

#### 3.3.5. Batch kinetic studies 

In batch kinetic adsorption experiments, ASMs (0.05 g) and methylene blue solution (50 mL, 200 mg/L) concentration were placed in a 125 mL flask and shaken by a shaker in a water bath to control the temperature. Every other period of time, 0.1 mL of MB solution was taken out, diluted to 10 mL with deionized water, and its concentration was determined at a wavelength of 665 nm with an UV-VIS spectrometer. 

## 4. Conclusions 

Anionic starch microspheres were prepared for the first time with chloroacetic acid as the anionic etherifying agent from neutral starch microspheres which were synthesized through a microemulsion crosslinking reaction. Anionic starch microspheres revealed comparatively uniform size distribution, better sphericity and dispersibility. FTIR spectroscopy results prove that ASMs were synthesized successfully.

The ASMs were used to adsorb methylene blue and displayed good adsorption performance. The adsorption process can be well described by a Langmuir isotherm with a maximum adsorption capacity of 667.7 mg/g. The temperature effect on the adsorption trend was insignificant; temperature variations do not significantly affect the overall decolorization performance. It can be concluded that 303 K may be the suitable adsorption temperature for MB adsorption by ASMs. The kinetic study showed that a pseudo-second-order kinetic models provided a better correlation of the experimental data in comparison with the pseudo-first-order model. This suggests that the rate-limiting step may be the chemical adsorption but not the mass transport.

## Figures and Tables

**Table 1:** Langmuir and Freundlich isotherm parameters for three different temperatures.

Temperature(K)	Langmuir equation				Freundlich equation		
	b(L/mg)	Q_m_(mg/g)	R^2^	ΔG^Ө^ (kJ/mol)	K_F_	n	R^2^
**293**	0.228	357.14	0.9969	−27.65	104.56	3.19	0.7589
**303**	0.119	666.67	0.9709	−26.96	80.6	1.5	0.9568
**313**	0.167	500	0.9909	−28.73	87.9	1.86	0.9536

**Table 2:** Kinetic parameters for three different temperatures.

Temperature(K)	Q_e-exp_	Pseudo-first-order			Pseudo-second-order		
		K_1_ (min^−1^)	Q_e-cal_ (mg/g)	R^2^	K_2_ (min^−1^)	Q_e-cal_ (mg/g)	R^2^
**293**	196.8	0.0475	25.81	0.8607	0.003	200	0.9998
**303**	196.56	0.054	17.24	0.9049	0.0058	200	1.0000
**313**	195.81	0.071	22.63	0.9323	0.0047	200	0.9998

**Table 3:** Comparison of the maximum monolayer adsorption of MB onto various adsorbents.

Adsorbents	Maximum monolayer adsorption capacity (mg/g)	References
Garlic peel	82.64	[33]
Rice husk	40.50	[34]
Raw beech sawdust	9.78	[35]
Oil palm trunk fibre	149.35	[36]
Broad bean peels	192.72	[37]
Activated rice husks	0.21	[38]
Date pits	80.31	[39]
Jute processing waste	22.47	[40]
ASMs	666.67	Present study
